# The KISS1/KISS1R Axis in Human Placentation: Molecular Mechanisms and Implications for Foetal Growth Restriction and Pre-Eclampsia

**DOI:** 10.3390/ijms27093748

**Published:** 2026-04-23

**Authors:** Elitsa Gyokova, Eleonora Hristova-Atanasova, Kamelia Dimitrova

**Affiliations:** 1 Department of Obstetrics and Gynecology, Faculty of Medicine, Medical University—Pleven, 5800 Pleven, Bulgaria; elitca.gaokova@mu-pleven.bg; 2 Clinic of Obstetrics and Gynecology, University Hospital “Saint Marina”, 5800 Pleven, Bulgaria; 3 Department of Social Medicine and Public Health, Faculty of Public Health, Medical University of Plovdiv, 4002 Plovdiv, Bulgaria; 4 Faculty of Medicine, Medical University—Pleven, 5800 Pleven, Bulgaria

**Keywords:** Kisspeptin-10, kisspeptin, kisspeptin receptor, foetal growth restriction, pre-eclampsia, placental dysfunction, trophoblast invasion, angiogenesis, spiral artery remodelling, angiogenic biomarkers

## Abstract

Pre-eclampsia and foetal growth restriction (FGR) are major pregnancy complications primarily driven by placental dysfunction, and remain leading causes of maternal and perinatal morbidity. Ultrasound imaging, Doppler studies, and angiogenic biomarkers like placental growth factor (PlGF) and soluble fms-like tyrosine kinase-1 (sFlt-1) constitute the main diagnostic modalities; however, these predominantly reflect established disease rather than early molecular disturbances underlying placentation. The identification of biomarkers directly associated with trophoblast signalling pathways has the potential to improve early risk stratification and enable mechanistic classifications. Kisspeptin signalling via its receptor (KISS1R) regulates trophoblast invasion, extracellular matrix remodelling, ERK1/2 activation, and angiogenic balance, thereby modulating spiral artery transformation. Kisspeptin-10 (KP-10), the minimal bioactive fragment of KISS1, is highly expressed in placental syncytiotrophoblasts and exerts its effects through the G-protein-coupled receptor KISS1R. Core features of early-onset FGR and pre-eclampsia (PE)—including defective placentation, maternal vascular malperfusion, and angiogenic imbalance—have been linked to dysregulation of this pathway. During normal gestation, maternal circulating kisspeptin concentrations rise exponentially. In contrast, pregnancies subsequently complicated by FGR or PE, particularly in the early gestation, are associated with reduced levels. However, the comparability of existing studies and their translational applicability are limited by a substantial methodological heterogeneity, including assay variability, gestational age dependence, and inadequate adjustment for maternal confounders. These limitations hinder robust conclusions regarding the role of kisspeptin in placental pathology. This review critically integrates molecular, pathophysiological, and clinical evidence relating to the role of KP-10 in placental dysfunction. The key question is whether KP-10 represents a mechanistic biomarker of trophoblast signalling dysfunction or merely a secondary marker of reduced placental mass; resolving this distinction is essential.

## 1. Introduction

Foetal growth restriction (FGR) is a condition in which a foetus fails to achieve its genetically determined growth potential, most commonly due to placental insufficiency. The American College of Obstetricians and Gynecologists and the Society for Maternal-Fetal Medicine define FGR as an estimated foetal weight or abdominal circumference below the 10th percentile for gestational age; however, they emphasise that FGR represents a pathological process rather than a purely statistical classification [1,2]. Accordingly, its clinical significance lies in underlying impaired placentation rather than biometric thresholds alone, necessitating a mechanistic understanding of placental development to improve risk stratification and diagnosis. Early-onset FGR, defined as diagnosis before 32 weeks’ gestation, is frequently associated with severe placental dysfunction, abnormal umbilical artery Doppler findings, and a high incidence of adverse perinatal outcomes, including pre-eclampsia (PE), preterm birth, and perinatal mortality. In contrast, late-onset FGR, typically diagnosed at or after 32 weeks, is generally milder, with fewer Doppler abnormalities, although it remains associated with impaired neurodevelopment and an increased risk of hypoxic injury [3,4,5]. These represent biologically distinct phenotypes. Early-onset FGR is strongly associated with defective trophoblast invasion and maternal vascular malperfusion, resulting from impaired spiral artery remodelling. This leads to placental ischaemia, infarction, distal villous hypoplasia, atherosis, and persistence of endovascular trophoblasts—lesions that are more prevalent in early-onset disease and correlate with abnormal Doppler findings and increased rates of PE, reflecting profound placental pathology and impaired maternal–foetal exchange [1,2,3,4,5]. At the molecular level, this phenotype is characterised by dysregulated trophoblast invasion, altered extracellular matrix turnover, oxidative stress, and angiogenic imbalance within the vascular endothelial growth factor (VEGF) and placental growth factor (PlGF) axis at the molecular level, consistent with a primary defect in placentation. In contrast, late-onset FGR (after 32–33 weeks) is more frequently associated with maternal cardiovascular maladaptation and relative placental insufficiency. Although placental structure is typically established, maternal haemodynamic adaptation fails to meet the increasing metabolic demands of late gestation, leading to relative hypoperfusion and reduced placental reserve. The clinical presentation is more heterogeneous, with a greater contribution from foetal vascular compromise, and placental pathology demonstrates fewer and less severe maternal vascular malperfusion lesions [1,6,7,8]. Doppler abnormalities are generally less pronounced and may reflect adaptive foetal redistribution rather than severe placental ischaemia.

Thus, early-onset FGR is primarily reflects abnormal placentation and maternal vascular malperfusion, whereas late-onset FGR reflects an inability of the maternal cardiovascular system to meet the demands of advanced gestation. This distinction is supported by maternal haemodynamic data, placental pathology, and clinical outcomes [1,2,4,5,6,7,8,9]. Recognising these mechanistic differences is essential when evaluating potential biomarkers, as early-onset disease is more likely to reflect upstream disturbances in placental regulatory signalling pathways. A foetus or neonate with an estimated foetal weight or birth weight below the 10th percentile is classified as small for gestational age (SGA), which does not necessarily indicate pathology [1,2,6]. In contrast, FGR represents a prenatal diagnosis of pathological growth restriction [1,7,8]. Accurate differentiation is critical, as FGR requires intensified surveillance and may necessitate timely delivery to reduce morbidity and mortality [8,9]. Placental dysfunction underlies both FGR and PE and results from abnormal trophoblast invasion and incomplete spiral artery remodelling. In normal pregnancy, extravillous trophoblasts transform spiral arteries into low-resistance, high-capacitance vessels. In PE and FGR, this process is incomplete, leading to maternal vascular malperfusion, placental ischaemia, oxidative stress, and systemic endothelial dysfunction [2,10,11,12,13,14,15,16]. These pathophysiological changes precede clinical manifestations by several weeks and provide a mechanistic basis for biomarker discovery. Angiogenic biomarkers, including placental growth factor (PlGF) and soluble fms-like tyrosine kinase-1 (sFlt-1), improve short-term risk stratification for PE by reflecting angiogenic imbalance [10,17,18,19,20,21,22,23]. However, their predictive performance for early identification of FGR—particularly late-onset disease—remains limited, as they primarily capture downstream vascular consequences rather than primary trophoblast regulatory pathways [10,20,21,22,23].

Kisspeptin-10 (KP-10) has emerged as a biologically plausible biomarker candidate. It is predominantly produced by placental syncytiotrophoblasts and increases markedly across gestation [24,25]. Reduced circulating kisspeptin levels have been reported in pregnancies complicated by FGR and PE, particularly in early gestation [24,25,26,27,28]. Beyond its role as a circulating marker, kisspeptin regulates trophoblast invasion, extracellular matrix remodelling, and angiogenic signalling through activation of its receptor KISS1R [24,29,30]. Through modulation of ERK1/2 phosphorylation, matrix metalloproteinase (MMP) activity, and VEGF expression, kisspeptin signalling may act upstream in the regulatory cascade governing spiral artery transformation and placental vascular development.

However, available human data are highly heterogeneous. Second-trimester studies generally report reduced kisspeptin levels preceding FGR, whereas third-trimester findings—particularly in late-onset disease—are inconsistent, with both decreased and increased concentrations reported [26,28]. These discrepancies likely reflect differences in assay methodology, gestational age adjustment, phenotype definition, and control for maternal confounders.

A major translational limitation is the absence of validated gestational age–specific reference ranges and standardised immunoassays. Given that kisspeptin concentrations rise exponentially during pregnancy, inadequate adjustment for gestational age may substantially distort observed associations [24,25,26]. Furthermore, the incremental value of kisspeptin within multimarker models remains unclear, and no prospective studies have directly compared its predictive performance with established angiogenic biomarkers such as PlGF or sFlt-1 in FGR.Whether circulating kisspeptin reflects primary dysregulation of trophoblast signalling or secondary changes in placental mass remains unresolved, particularly in late-onset FGR, where divergent findings suggest potential compensatory mechanisms. Although PE and FGR are multifactorial disorders, increasing evidence suggests that the disruption of discrete signalling pathways may act as molecular bottlenecks within complex placental networks.

One such regulatory axis is the kisspeptin (KISS1/KISS1R) system, which functions upstream in the control of trophoblast invasion and vascular remodelling. Perturbation of this pathway may phenocopy monogenic defects within otherwise polygenic placental disease.

Accordingly, understanding the regulatory architecture of the KISS1/KISS1R axis is essential for the development of translational biomarkers and mechanistic classification frameworks. The aim of this review is to critically evaluate the molecular role of KISS1/KISS1R signalling in placental development, synthesise available human evidence on circulating KP-10 across gestation in FGR with or without PE, and propose a mechanistic framework to guide future prospective validation studies and biomarker integration prior to clinical implementation.

## 2. Materials and Methods

This narrative review was conducted using a structured approach informed by the framework described by Templier and Paré, with the objective of critically synthesising mechanistic and clinical evidence relating to KP-10 in placental dysfunction, FGR, and PE. A narrative design was selected to enable integration of heterogeneous evidence spanning molecular biology, translational research, clinical observational studies, and biomarker performance data, which cannot be fully captured using conventional systematic review methodologies. To enhance transparency, methodological rigour, and critical appraisal, the review process was guided by the SANRA (Scale for the Assessment of Narrative Review Articles) standard [31].

A comprehensive literature search was conducted across the Cochrane Library (Wiley, Hoboken, NJ, USA), Scopus (Elsevier, Amsterdam, Netherlands), Google Scholar (Google LLC, Mountain View, CA, USA), MEDLINE/PubMed (National Library of Medicine, Bethesda, MD, USA), and Web of Science (Clarivate Analytics, London, UK). The search strategy incorporated terms related to kisspeptin biology and placental disease, including “kisspeptin,” “kisspeptin-10,” “KISS1,” “KISS1R,” “placentation,” “trophoblast invasion,” “spiral artery remodelling,” “angiogenesis,” “foetal growth restriction,” “intrauterine growth restriction,” and “pre-eclampsia.” The initial PubMed search strategy included the following terms within Title/Abstract fields: (“kisspeptin” OR “KISS1” OR “kisspeptin-10”) AND (“foetal growth restriction” OR “IUGR” OR “pre-eclampsia” OR “placental dysfunction”). Boolean operators were applied to optimise sensitivity and specificity, with incorporation of Medical Subject Headings (MeSH) where appropriate.

The search was restricted to human studies published between January 2000 and February 2026, reflecting the period of substantial expansion in placental kisspeptin research and clinical biomarker evaluation. Animal and mechanistic studies were included selectively when directly relevant to trophoblast biology or placental signalling pathways.

Eligible sources comprised peer-reviewed original research articles, prospective and retrospective cohort studies, case–control studies, cross-sectional analyses, in vitro and ex vivo mechanistic investigations, systematic reviews, narrative reviews, and international clinical guidelines addressing placental dysfunction, angiogenic biomarkers, or kisspeptin signalling in pregnancy. Conference abstracts, isolated case reports, non–peer-reviewed material, and non-English publications were excluded to maintain methodological consistency and interpretability.

Given the recognised heterogeneity in biomarker research, particular attention was directed towards methodological variables, including assay type (e.g., ELISA versus other immunoassays), detection limits, intra- and inter-assay variability, gestational age adjustment, use of multiples of the median (MoM), sample matrix (serum versus plasma), and adjustment for maternal confounders such as body mass index, smoking status, and metabolic comorbidities. These analytical factors were qualitatively incorporated into the synthesis of reported diagnostic performance metrics.

The evidence was synthesised narratively and organised into the following thematic domains: (1) molecular mechanisms of KISS1/KISS1R signalling in placentation; (2) pathophysiological integration with angiogenic and vascular pathways; (3) human observational evidence across gestation; (4) methodological heterogeneity and assay limitations; and (5) translational implications and gaps in clinical integration. Emphasis was placed on mechanistic plausibility, consistency across gestational windows, phenotype-specific patterns (early- versus late-onset FGR and PE subtypes), and comparison with established angiogenic biomarkers.

A formal meta-analysis was not undertaken due to substantial heterogeneity in assay methodologies, gestational age correction, outcome definitions, and study populations. Instead, a critical interpretive synthesis approach was employed to identify convergent and divergent findings and to highlight areas requiring prospective validation.

## 3. Biology of Kisspeptin Signalling Relevant to Placentation

In the human placenta, the *KISS1* gene is highly expressed, particularly within syncytiotrophoblasts. It encodes a precursor peptide that is cleaved into several biologically active isoforms, including kisspeptin-54, kisspeptin-14, kisspeptin-13, and the shortest, KP-10. These isoforms share a common C-terminal decapeptide sequence (KP-10), which represents the minimal sequence required for full activation of the kisspeptin receptor (KISS1R). KISS1R, a G-protein-coupled receptor, is widely expressed in placental tissues, such as cytotrophoblasts, syncytiotrophoblasts, extravillous trophoblasts, and placental blood vessels [29,32,33,34,35,36]. The expression of KISS1 and KISS1R in the placenta is dynamic throughout gestation, protein levels paralleling the exponential rise in maternal circulating kisspeptin concentrations [29,34,35,37]. Functionally, KISS1 and KISS1R are both involved in the regulation of trophoblast invasion and migration. Kisspeptin signalling functions as a negative regulator, limiting excessive trophoblast invasion and thereby contributing to normal placental development and function [30].

Among the isoforms, KP-10 is most potent activator of KISS1R and is present in both the placenta and maternal circulation [33,34,36]. Although kisspeptin-54 is also abundant, all isoforms exert their biological effects via KISS1R activation [22,34]. The biological plausibility of kisspeptin as a placental biomarker is supported by its placental origin, gestational dynamics, and direct regulatory role in trophoblast behaviour and placental angiogenesis [29,36].

Kisspeptin signalling exerts inhibitory effects on trophoblast migration and invasion, thereby modulating spiral artery remodelling. While kisspeptin is predominantly expressed in syncytiotrophoblasts, KISS1R is localised to cytotrophoblasts and extravillous trophoblasts. Activation of this pathway downregulates multiple matrix metalloproteinases (MMP-1, -2, -3, -7, -9, -10, and -14) and vascular endothelial growth factor A (VEGF-A), while upregulating tissue inhibitors of metalloproteinases (TIMP-1 and TIMP-3). This coordinated regulation limits trophoblast penetration into the maternal decidua and spiral arteries [38,39]. This inhibitory effect is crucial for fine-tuning the depth of trophoblast invasion and ensuring controlled spiral artery remodelling. Excessive inhibition, as observed in placentas from early-onset PE with altered KISS1 and/or KISS1R expression is associated with shallow trophoblast invasion and defective spiral artery transformation [35,40]. Evidence for KISS1/KISS1R dysregulation is considerably stronger in early-onset PE, whereas late-onset disease demonstrates more heterogeneous and less consistent molecular patterns, suggesting phenotype-specific involvement of kisspeptin signalling. Conversely, genetic or functional loss of kisspeptin signalling leads to excessive trophoblast invasion and abnormal placental architecture, as demonstrated in Kiss1r knockout models [32,41]. TGF-β1 has been shown to upregulate KISS1 expression via ERK1/2-dependent pathways, further reinforcing the inhibitory control over trophoblast invasion [40]. In addition, kisspeptin enhances extravillous trophoblast adhesion to type I collagen through PKC- and ERK1/2-dependentpathways, indicating role of integrin-mediated adhesion and cytoskeletal organization [42]. Collectively, these findings underscore the importance of balanced KISS1/KISS1R signalling in maintaining appropriate invasion depth and structural integrity at the maternal–foetal interface [24,29,43].

At the intracellular level, KISS1R (also known as GPR54) primarily couples to Gαq/11 proteins, activating phospholipase C (PLC) and inducing hydrolysis of PIP_2_ into IP_3_ and DAG [44,45]. IP_3_ induces intracellular Ca^2+^ release followed by sustained calcium influx, while DAG activates protein kinase C (PKC). These signals converge on MAPK/ERK pathway, with rapid ERK1/2 phosphorylation after kisspeptin stimulation [38,42]. Downstream, KISS1R signalling suppresses MMP-1, -2, -3, -7, -9, -10, and -14 and upregulates TIMP-1 and TIMP-3. This regulatory balance limits trophoblast invasion and contributes to controlled spiral artery remodelling [37,38]. ERK1/2-dependent feedback involving GSK3β and FAK modulates β-catenin signalling and integrin-mediated adhesion to type I collagen, reinforcing controlled placental anchoring [42,46].

**Figure 1:**
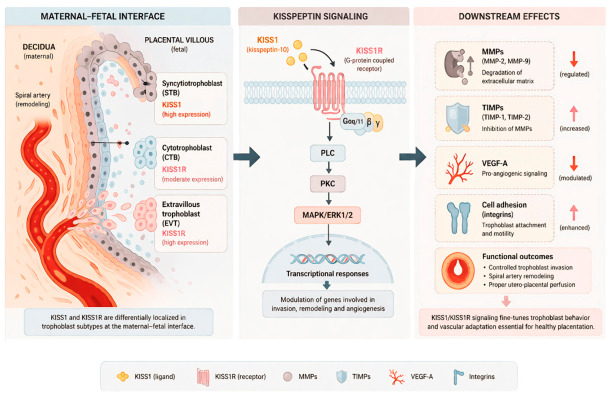
KISS1/KISS1R signalling at the maternal–foetal interface. Schematic representation of kisspeptin signalling in placental tissue, illustrating trophoblast subtypes (syncytiotrophoblast, cytotrophoblast, extravillous trophoblast), localisation of KISS1 and KISS1R, and downstream signalling pathways. Activation of KISS1R triggers Gαq/11–PLC–PKC–ERK1/2 signalling, leading to modulation of matrix metalloproteinases (MMPs), tissue inhibitors of metalloproteinases (TIMPs), and vascular endothelial growth factor (VEGF), thereby regulating trophoblast invasion, adhesion, and spiral artery remodelling.

The spatial organisation and downstream signalling effects of KISS1/KISS1R at the maternal–foetal interface are summarised in Figure 1.

KISS1R signalling also attenuates VEGF-A expression and interacts with TGF-β pathways. TGF-β1 upregulates kisspeptin via ERK1/2-dependent mechanisms, establishing a feed-forward inhibitory loop that restrains excessive trophoblast invasion [47,48]. Through the integrated Gαq/11–PLC–PKC–ERK signalling axis, kisspeptin regulates extracellular matrix remodelling, angiogenic balance, and vascular adaptation, positioning KISS1R as a central regulator of placental invasion depth. The apparent divergence between increased placental KISS1/KISS1R expression and reduced maternal circulating kisspeptin levels reported in PE suggests compartment-specific dysregulation rather than simple downregulation of synthesis, highlighting the complexity of local versus systemic signalling dynamics.

A structured overview of the principal molecular mechanisms through which KISS1/KISS1R signalling regulates placentation is presented below (Table 1).

Collectively, KISS1/KISS1R signalling integrates extracellular matrix remodelling, kinase activation, angiogenic modulation, and trophoblast adhesion, positioning it as a nodal regulator of early placental architecture rather than a single-pathway invasion inhibitor. The convergence of MMP/TIMP balance, ERK1/2 signalling, and VEGF modulation supports the concept that dysregulation of these pathways may directly influence placental vascular architecture and maternal-foetal exchange.

Angiogenesis and vascular regulation are essential for placental development, ensuring adequate formation and remodelling of the vascular network that supports foetal growth. Placental angiogenesis is driven by the VEGF axis, including VEGF-A, PlGF, VEGFR1, and VEGFR2, which regulate endothelial proliferation, migration, and branching morphogenesis [49,50,51,52,53]. This system is tightly regulated: VEGF-A and PlGF stimulate angiogenesis and vasodilation, while anti-angiogenic mediators such sFlt-1 and VEGF-A165b antagonise these effects, modulating endothelial barrier integrity and vascular permeability [54,55].

Imbalance within this system leads to impaired perfusion, increased vascular resistance, and placental ischaemia—hallmarks of FGR and PE [51,54,56,57,58]. Endothelial function is further modulated by nitric oxide and downstream vasodilatory mediators, which reduce vascular tone and facilitate uteroplacental blood flow [58,59,60].

Hormonal and metabolic pathways further interact with placental dysfunction in FGR and PE. Disrupted placental steroidogenesis, altered glucose and fatty acid metabolism, oxidative stress, leptin dysregulation, and aberrant cortisol/aldosterone signalling contribute to impaired trophoblast function, vascular maladaptation, and reduced placental efficiency [61,62,63,64,65,66,67]. These processes align with an integrated maternal cardiovascular-placental-foetal model, in which maladaptation in any axis can precipitate placental insufficiency and adverse pregnancy outcomes [16,68].

Transcriptional and epigenetic regulation of the human *KISS1* gene critically determines placental-specific expression patterns and likely contributes to phenotype-specific differences between early- and late-onset placental disease. *KISS1* promoter activity is responsive to hypoxia-inducible factors (HIFs), steroid hormones—including oestrogens and glucocorticoids—and transforming growth factor-β (TGF-β) signalling pathways, all of which are dynamically modulated within the placental microenvironment [29]. Under hypoxic conditions in early gestation HIF activation may enhance or repress *KISS1* transcription depending on cellular context, thereby influencing trophoblast invasion depth and spiral artery remodelling [29].

Glucocorticoids regulate placental *Kiss1* and Kiss1r expression in a gestational-age-dependent manner [69]. Early glucocorticoid exposure has been associated with suppression of trophoblast proliferation and invasion, whereas later reductions in signalling correlate with impaired placental growth and features of growth restriction [69]. TGF-β signalling further upregulates *KISS1* expression and inhibits trophoblast invasion through ERK1/2-dependent pathways rather than canonical SMAD signalling, reinforcing invasion-restrictive regulatory loops within the placental environment [47].

Epigenetic mechanisms provide an additional layer of regulation. Genome-wide epigenomic analyses demonstrate dynamic remodelling of DNA methylation patterns and histone modifications during placental maturation [70,71]. Progressive promoter methylation and modulation of activating histone marks such as H3K27ac are associated with physiological trophoblast differentiation. In contrast, aberrant chromatin states—including altered acetylation patterns and immature epigenomic signatures—have been described in severe PE, resulting in dysregulated transcriptional programmes that may include the KISS1 axis [70,72]. Evidence from neuroendocrine systems further supports the plausibility of context-dependent regulation of KISS1 expression [73].

These epigenetic alterations align with the distinct molecular landscapes observed in early-onset placental disease—characterised by hypoxia, defective trophoblast invasion, and developmental immaturity—versus late-onset phenotypes, which display more compensatory, metabolically adaptive, and stress-responsive placental signatures [71]. Such divergence suggests that gestational timing and microenvironmental context critically shape KISS1/KISS1R regulatory dynamics.

Notably, experimental disruption of the KISS1/KISS1R signalling axis recapitulates impaired trophoblast invasion, abnormal vascular remodelling, and altered placental architecture in both cellular and animal models [32,41]. Placental tissue analyses further demonstrate altered KISS1 and KISS1R protein expression in pregnancies complicated by PE and metabolic disease [74]. Functional perturbation of this single-gene signalling axis—whether driven by transcriptional dysregulation or epigenetic modification—may therefore produce tissue-level effects analogous to monogenic disruption, even in the absence of inherited pathogenic variants. This conceptual framework positions the KISS1/KISS1R system as a molecular vulnerability node in placental disease, linking environmental stressors, regulatory instability, and adverse pregnancy phenotypes such as FGR and PE.

## 4. Pathophysiological Bridge: From Abnormal Placentation to FGR (±PE)

As outlined in Section 3, KISS1/KISS1R signalling regulates trophoblast invasion through modulation of MMP/TIMP balance, ERK1/2 activation, and angiogenic pathways. Early placental dysfunction is characterised by defective trophoblast invasion and inadequate spiral artery remodelling, which leads to impaired uteroplacental perfusion, placental ischaemia, oxidative stress, and endoplasmic reticulum stress. This phenotype typically presents before 32–34 weeks’ gestation and is strongly associated with early-onset FGR and early-onset PE. Clinically, early FGR often features severe growth restriction, abnormal umbilical artery Doppler findings (including absent or reversed end-diastolic flow), and a high incidence of PE. Placental pathology in early FGR and PE shows pronounced maternal vascular malperfusion, including infarcts, distal villous hypoplasia, atherosis, reduced placental efficiency, and extensive histopathological and molecular abnormalities [11,75,76,77,78].

At the molecular level, early placental pathology reflects a shift toward anti-angiogenic dominance, oxidative stress amplification, and dysregulated trophoblast differentiation, creating a feed-forward cycle of hypoperfusion and inflammatory activation [11,15]. These processes precede clinical manifestations by weeks to months and ultimately contribute to the maternal endothelial dysfunction characteristic of PE [79,80].

In contrast, late placental dysfunction (≥32–34 weeks) is more often associated with maternal cardiovascular or metabolic maladaptation rather than primary defects in early trophoblast invasion. Late-onset FGR and late-onset PE are generally milder, with less severe growth restriction, fewer Doppler abnormalities, and lower rates of adverse perinatal outcomes. Correspondingly, placental lesions are less frequent and less severe, often reflecting adaptive or senescent processes rather than profound ischaemic injury [16,75,76,77,78,79].

FGR with PE and isolated FGR share placental disease as a common pathophysiological substrate but differ in clinical phenotype and Doppler characteristics.

In FGR complicated by PE, cases are more often early-onset (<32 weeks) and present with maternal hypertension and proteinuria. These pregnancies demonstrate more extensive maternal vascular malperfusion, infarction, and atherosis, resulting in higher rates of prematurity and adverse perinatal outcomes. Doppler findings typically include elevated uterine and umbilical artery pulsatility indices and absent or reversed end-diastolic flow, often progressing to ductus venosus abnormalities in severe cases [1,11,13,81,82,83,84,85]. Such haemodynamic deterioration reflects advanced placental insufficiency and foetal cardiovascular adaption.

By contrast, isolated FGR (without PE) is more commonly late-onset (>32 weeks) and occurs in the absence of maternal hypertension. Placental lesions are generally less severe, and Doppler abnormalities may be milder. Umbilical artery Doppler may be normal or only slightly abnormal, and uterine artery Doppler is less frequently pathological. Middle cerebral artery Doppler may demonstrate “brain-sparing”, but progression to severe venous Doppler abnormalities are less common than in FGR with PE [1,4,9,82,83,85,86,87].

Thus, the timing, severity, and systemic maternal response to abnormal placentation determine whether the phenotype manifests as isolated FGR, PE, or the combined FGR–PE syndrome. Severe early placental maldevelopment favours the combined phenotype, whereas later or milder dysfunction may present as isolated FGR.

Within this pathophysiological cascade, KP-10 is biologically positioned upstream as a regulator of trophoblast invasion and placental development. [29,30]. Dysregulation of this pathway contributes to impaired placental development and uteroplacental perfusion, thereby contributing to FGR and PE [29,30,40,41,88].

From a mechanistic perspective, altered kisspeptin signalling may represent one of the earliest detectable molecular perturbations within the placental developmental programme, preceding overt angiogenic imbalance and clinical disease.

**Figure 2:**
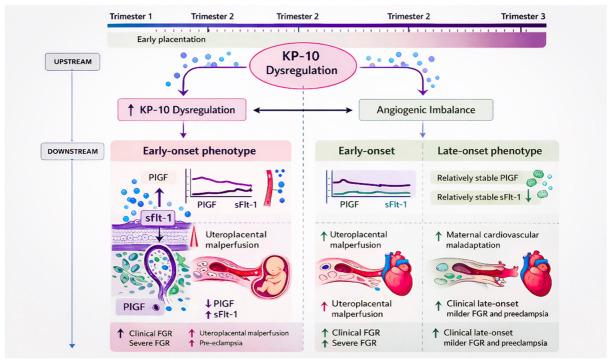
Temporal relationship between KP-10 dysregulation and angiogenic imbalance across gestation.

The proposed temporal relationship between early kisspeptin dysregulation and downstream angiogenic imbalance across gestation is illustrated in Figure 2.

Conceptual model illustrating the position of kisspeptin (KP-10) within the placental disease cascade. Early dysregulation of KP-10 is proposed to occur upstream during placentation, preceding measurable angiogenic imbalance. In early-onset phenotypes, reduced KP-10 is associated with decreased PlGF, increased sFlt-1, severe uteroplacental malperfusion, and increased risk of pre-eclampsia and severe foetal growth restriction. In late-onset phenotypes, angiogenic alterations are more heterogeneous and may reflect compensatory placental and maternal cardiovascular responses, resulting in milder clinical manifestations.

Circulating kisspeptin levels increase markedly across normal gestation and reflect placental functional capacity [26]. Several studies have demonstrated reduced maternal kisspeptin concentrations in pregnancies that subsequently develop FGR or PE [89,90], supporting its role as an early biomarker of placental dysfunction rather than a late marker of established disease.

However, interpretation of circulating kisspeptin requires careful phenotype stratification. Emerging evidence indicates gestational-age–dependent and phenotype-specific patterns. In a large prospective cohort with serial trimester sampling, third-trimester kisspeptin levels were lower in FGR, with decreasing odds of FGR per unit increase in kisspeptin concentration [26]. In contrast, a case–control study reported higher kisspeptin levels in late-onset FGR [28]. Similarly, meta-analyses suggest reduced circulating kisspeptin in PE overall [90], whereas elevated levels have been reported in late-onset PE compared with gestational age–matched controls [91].

These divergent findings suggest that circulating kisspeptin does not reflect a uniform biological signal but rather integrates placental endocrine activity, trophoblast invasion status, and maternal cardiovascular adaptation. In late-onset phenotypes, increased circulating levels may represent compensatory endocrine activation of a structurally established but functionally stressed placenta, rather than primary failure of early invasion.

Of particular relevance, placental expression studies frequently demonstrate increased KISS1 and/or KISS1R expression in preeclamptic placentas despite reduced maternal circulating kisspeptin concentrations [35,74]. This apparent expression–circulation paradox remains unresolved and may involve impaired peptide processing, altered secretion from dysfunctional syncytiotrophoblasts, increased local degradation, or compensatory receptor upregulation.

Taken together, abnormal placentation initiates a temporally dynamic and phenotype-specific cascade of molecular, vascular, and haemodynamic events culminating in FGR with or without maternal PE. KP-10 occupies a mechanistically plausible upstream position within this cascade, linking early trophoblast invasion control to downstream angiogenic imbalance and clinical phenotype.

Accordingly, kisspeptin should not be interpreted as a static surrogate of placental mass, but rather as a dynamic indicator of trophoblast–vascular crosstalk within a phenotype-dependent pathophysiological continuum.

## 5. Human Evidence: Maternal Circulating KP-10 Across Gestation

Maternal circulating kisspeptin, including KP-10, increases exponentially across normal pregnancy, reflecting placental synthesis and syncytiotrophoblast secretion [24,25,30]. Gestational age is the principal physiological determinant of circulating concentrations and must be rigorously accounted for in all comparative analyses [24,25]. Additional variability arises from the measurement of different kisspeptin isoforms (KP-10 versus longer peptides such as KP-54) as well as the lack of harmonisation regarding free versus total circulating fractions.

### 5.1. First Trimester

Reduced first-trimester kisspeptin concentrations have been associated with subsequent FGR and PE [43,89,90]. In obese pregnancies, kisspeptin demonstrated moderate predictive performance for PE, with an AUC of 0.80, sensitivity 85.7% and specificity 71.4% [92]. However, external validation in broader obstetric populations remains limited.

Within the same gestational window, PlGF demonstrates superior predictive performance. When measured at ≥10 weeks’ gestation and combined with maternal factors, PlGF increased the detection rate for preterm PE from 31.3% to 56.3% at a 10% screen-positive rate [93,94]. In contrast, PAPP-A (pregnancy-associated plasma protein A) shows more limited discriminatory capacity; PAPP-A < 5th centile is associated with OR 2.08 for SGA and OR 1.94 for PE, but with modest likelihood ratios (LR+ 1.96; LR− 0.93) [95]. Combined PlGF and ADMA (asymmetric dimethylarginine) achieved an AUC of 0.902 for PE prediction [96].

Importantly, kisspeptin has primarily been evaluated as an early screening or risk-stratification marker, whereas angiogenic biomarkers have established diagnostic and short-term rule-out utility, representing fundamentally different clinical applications.

### 5.2. Second Trimester

In the second trimester, kisspeptin demonstrates modest and phenotype-dependent differences. The greatest separation between affected and unaffected pregnancies appears at 16–20 weeks’ gestation, with attenuation thereafter [91,97]. However, directionality is inconsistent between early- and late-onset phenotypes, limiting the definition of robust diagnostic thresholds.

By contrast, PlGF combined with foetal Doppler indices demonstrates substantially stronger discriminatory performance for early placental disease. In early-onset SGA, combined angiogenic and Doppler-based models achieved an AUC of 0.866 for prediction of composite adverse perinatal outcomes [98].

### 5.3. Third Trimester

In the third trimester, the diagnostic performance of kisspeptin is limited. In late-onset FGR, a proposed cut-off of 30.32 pg/mL yielded a positive predictive value of 64.6% and a negative predictive value of 87.5%, but with considerable overlap between affected and unaffected groups [28]. In hypertensive disorders, increased third-trimester concentrations have also been reported [97], further reducing specificity for placental insufficiency.

By contrast, the sFlt-1/PlGF ratio demonstrates markedly superior and clinically validated performance. An AUC of 0.92 has been reported for severe PE [99]. A ratio > 38 provides sensitivity 80%, specificity 92%, and NPV 99.3% for PE within 7 days, while ≥74 achieves sensitivity 87.7% and specificity 97.0% for early-onset PE [100,101]. Additionally, PlGF < 100 pg/mL demonstrates a sensitivity of 96% and NPV of 98% for PE requiring delivery within 14 days [102]. Furthermore, the sFlt-1/PlGF ratio reliably distinguishes pathological FGR from constitutionally small foetuses (SMD 0.58) [103]. A comparative overview of biomarker performance across gestation is provided in Table 2.

Interpretation of circulating kisspeptin is constrained by assay heterogeneity, peptide instability, and inter-assay variability, as well as a lack of standardised calibration systems [25,104,105,106]. Additional complexity arises from phenotype-specific directionality, with reductions observed in FGR but increases reported in some late-onset hypertensive phenotypes [28,91,97]. Overall, standardised effect sizes remain modest with considerable overlap between affected and unaffected populations [90]. Validated gestational-age-specific diagnostic thresholds are not yet established.

Conceptually, kisspeptin appears to reflect upstream trophoblast differentiation and placental development processes, whereas angiogenic biomarkers more directly reflect downstream endothelial dysfunction and established placental stress.

The biological plausibility of kisspeptin as a marker of abnormal placentation is strong; however, biological relevance does not necessarily translate into clinical discriminative performance. A key unmet clinical need remains the identification of biomarkers capable of predicting placental dysfunction prior to the onset of angiogenic imbalance and overt clinical disease. Whether kisspeptin can contribute meaningfully to early stratification within multimarker predictive models remains an open question requiring prospective validation.

## 6. Methodological Heterogeneity: Why Results Diverge

Divergent findings regarding circulating KP-10 in FGR and PE arise from multiple sources of heterogeneity, including analytical, pre-analytical, biological, clinical, and statistical factors. These domains interact and collectively limit reproducibility, cross-study and clinical translation. In addition, KP-10 concentrations vary substantially across gestation, reflecting dynamic changes in placental development and endocrine activity. The absence of standardised trimester-specific reference ranges further limits comparability across studies and complicates clinical interpretation. These domains interact and collectively limit reproducibility, cross-study comparability, and clinical translation [24,25,26,43].

At the analytical level, substantial inter-assay variability characterises the kisspeptin literature. Immunoassays differ in antibody specificity, calibration systems, detection limits, and cross-reactivity with kisspeptin isoforms. Although all biologically active isoforms (KP-10, KP-13, KP-14, KP-54) share the common C-terminal decapeptide sequence required for KISS1R activation [33,34], assay performance may vary depending on epitope recognition and peptide length detection. A validated platform reported a detection limit of 24 pg/mL (0.024 ng/mL) with intra-assay and inter-assay coefficients of variation of 5.1% and 8.6%, respectively [90,107]. However, such performance characteristics are not standardised across platforms, and laboratory-developed assays frequently lack external calibration [24,25,26]. Variability in absolute concentrations and detection thresholds has been consistently identified as a major contributor to inter-study inconsistency [24,25,43].

Pre-analytical factors further contribute to inconsistent findings. KP-10 is susceptible to proteolytic degradation, and plasma—particularly EDTA-anticoagulant samples—is generally preferred over serum due to reduced ex vivo proteolysis [24,25,26]. This is especially relevant for short peptides such as KP-10, where even minor variations in handling may significantly alter measured concentrations.

Critical variables—including anticoagulant type, time to centrifugation, use of protease inhibitors, storage temperature (≤−70 °C), freeze–thaw cycles, and tube material—are inconsistently reported across studies. Adsorption to collection surfaces and peptide instability at room temperature or 4 °C can lead to falsely low concentrations if processing is delayed [25,26].

Gestational age represents the dominant biological determinant of circulating kisspeptin. Plasma concentrations rise exponentially across normal pregnancies [25,26,27], and a strong correlation with gestation has been demonstrated (r^2^ = 0.57, *p* < 0.0001) [27,29]. Without strict gestational-age matching or statistical correction, observed differences may reflect physiological variation rather than pathological deviation [24,25]. Longitudinal cohort studies demonstrate trimester-specific and phenotype-dependent associations, including lower third-trimester levels in FGR and higher levels in hypertensive disorders [26,27]. These findings underscore the importance of gestational context in interpreting circulating KP-10.

Early- and late-onset PE demonstrate distinct biological profiles [10,75]. Higher circulating kisspeptin has been reported in late-onset PE, whereas no significant difference was observed in early-onset disease [9,91]. Similarly, third-trimester levels have been reported as higher in late-onset FGR in one cohort [28], contrasting with prospective studies demonstrating overall reduced levels in FGR [26,27]. Meta-analytic evidence suggests an overall lower circulating kisspeptin in PE (SMD −0.68, 95% CI −1.04 to −0.32) [90,108]. In contrast, placental tissue studies frequently demonstrate increased *KISS1* and *KISS1R* expression in preeclamptic placentas [35,38,40,88,108], despite reduced maternal circulating concentrations.

Circulating kisspeptin correlates with maternal weight, BMI, insulin resistance, and leptin levels [109]. Elevated urinary kisspeptin immunoreactivity has also been described during pregnancy [110]. In obese and gestational diabetic pregnancies, altered placental kisspeptin signalling has been reported [40], and correlation patterns between obese and non-obese populations [111]. Maternal cardiovascular maladaptation and endocrine alterations interact with placental dysfunction [64,68], potentially modifying circulating kisspeptin independently of placental vascular pathology.

FGR represents a pathological prenatal diagnosis based on ultrasound biometry and Doppler findings [1,2,9], whereas SGA refers to a statistical definition based on birthweight. Failure to distinguish constitutionally small fetuses from true FGR introduces substantial misclassification bias and dilutes observed biomarker effect sizes [8,82].

Population-specific variability in KP-10 signalling remains insufficiently characterised and represents an additional source of heterogeneity in the current literature. Emerging evidence suggests that metabolic status, BMI distribution, insulin resistance, and endocrine profiles may influence circulating kisspeptin concentrations and placental signalling dynamics [40,109,111]. However, the direction and magnitude of these associations appear context-dependent and may differ between populations. Sex-specific and age-related differences in kisspeptin physiology may further complicate interpretation, reflecting broader endocrine regulatory mechanisms that may interact with placental function during pregnancy. Preliminary data also suggest that placental KISS1/KISS1R expression and circulating kisspeptin levels may be modified by maternal metabolic conditions such as obesity and gestational diabetes, although findings remain inconsistent [40,74]. Critically, most available studies are derived from relatively homogeneous populations, with limited representation of diverse ethnic groups. Differences in baseline metabolic and cardiovascular profiles across populations may contribute to variability in reported associations between KP-10 and placental dysfunction [11,111]. The absence of ethnicity-stratified analyses and population-specific reference ranges represents a significant limitation for clinical translation. Future studies should incorporate diverse cohorts and apply stratified analyses accounting for ethnicity, BMI, metabolic status, and cardiovascular adaptation. Establishing population-specific reference ranges and standardised analytical approaches will be essential to improve the generalisability and clinical utility of KP-10 as a biomarker of placental pathology.

Many studies are retrospective, single-centre, and based on single time-point sampling with limited external validation [24,25]. Overlapping distributions and modest pooled effect sizes further limit discriminative performance [90]. Prospective longitudinal cohort studies with gestational-age correction phenotype stratification, and multivariable modelling are therefore essential prior to clinical implementation (Table 3).

## 7. Clinical Translation: Where Could KP-10 Add Value?

KP-10 occupies a biologically distinct position within the placental disease cascade and may therefore offer complementary value to established angiogenic biomarkers in FGR and PE. As a syncytiotrophoblast-derived peptide whose circulating concentrations rise exponentially during normal gestation, KP-10 reflects trophoblast functional integrity rather than downstream endothelial stress. Reduced first- and second-trimester concentrations have been associated with the subsequent development of FGR and PE [24,26,89,92], consistent with its regulatory role in trophoblast invasion and spiral artery remodelling [29]. Mechanistically, KP-10 represents an upstream marker of placental developmental signalling, whereas PlGF and sFlt-1 reflect later angiogenic disequilibrium and maternal endothelial dysfunction [17,18].

This temporal distinction is clinically relevant. Angiogenic markers demonstrate high diagnostic and short-term predictive utility in the second and third trimesters, particularly for ruling out or confirming established disease. By contrast, KP-10 may hold greater value during early placentation, when structural and haemodynamic abnormalities are not yet detectable. However, available data indicate only moderate discriminatory performance. Early pregnancy studies report AUC values for PE prediction approximate 0.80 [92], with sensitivity 85.7% and specificity 71.4% at around 16 weeks in one cohort [26]. In late-onset FGR, reported positive and negative predictive values are 64.6% and 87.5%, respectively [28]. Meta-analytic synthesis demonstrates a standardised mean difference of −0.68 (95% CI −1.04 to −0.32) [90], reflecting statistically significant but clinically overlapping distributions. Collectively, these findings support biological relevance but limit standalone diagnostic utility. Accordingly, the most plausible translational pathway for KP-10 lies within multimarker screening frameworks rather than isolated test. Contemporary risk algorithms integrating maternal characteristics, mean arterial pressure, uterine artery Doppler, PlGF, and sFlt-1 achieve AUC values approaching 0.89 with sensitivity and specificity exceeding 80% [23,112]. The Foetal Medicine Foundation competing-risks model represents the most extensively validated paradigm [80]. Within such integrated models, each biomarker captures a distinct biological domain: Doppler indices quantify uteroplacental haemodynamics, angiogenic factors reflect endothelial stress, and maternal haemodynamics capture systemic vascular adaptation. KP-10 may theoretically contribute a developmental signalling dimension, particularly in the first trimester before angiogenic imbalance becomes apparent. However, its incremental predictive value remains unproven, and no studies to date have demonstrated improved performance when KP-10 is added to established screening models, including formal reclassification analyses [113].

Early-onset FGR, characterised by severe placental malperfusion, abnormal umbilical artery Doppler findings, and high-grade vascular lesions [9,22,76,77,114,115], may represent the phenotype most closely aligned with upstream trophoblast dysregulation and thus the most biologically coherent target for KP-10-based early risk stratification. In contrast, late-onset FGR demonstrates milder and more heterogeneous placental pathology with predominant cerebroplacental redistribution [5,9,71,77,115,116], potentially explaining inconsistent third-trimester KP-10 findings.

Several barriers currently preclude clinical adoption. Assay heterogeneity, limited standardisation, absence of certified reference materials, and lack of validated gestational age–specific reference ranges significantly limit reproducibility [24,25,26,92]. Pre-analytical instability and isoform cross-reactivity further complicate interpretation. In addition, confounding by body mass index, metabolic status, smoking, and ethnicity necessitates robust multivariable adjustment for valid effect estimation [26,42,90]. Without harmonised methodology and external validation cohorts, clinically reliable thresholds cannot be established. Taken together, KP-10 should not be viewed as a replacement for PlGF or sFlt-1 nor as a diagnostic biomarker of established placental insufficiency. Rather, its potential lies in early developmental risk stratification as part of a biologically layered, multimodal screening strategy. Future research must determine whether the incorporation of KP-10 into first-trimester competing-risks models improves detection rates for early-onset FGR and preterm PE beyond current standards and whether such improvement translates into meaningful clinical benefit through targeted prophylaxis and surveillance. Until such evidence is available, KP-10 remains a promising but investigational biomarker within the translational continuum of placental disease.

## 8. Research Gaps and an Agenda for Next Studies

Despite compelling biological plausibility, the principal limitations in KP-10 research are methodological rather than mechanistic. Current evidence consistently demonstrates associations between altered circulating KP-10 concentrations and placental dysfunction; however, the lack of analytical harmonisation, inadequate phenotypic stratification, and limited prospective validation preclude definitive conclusions regarding clinical utility. The central translational challenge is therefore not whether KP-10 is biologically relevant but whether its measurement can be standardised and shown to provide incremental predictive value beyond established screening paradigms.

Analytical heterogeneity remains the most immediate barrier. Studies employ diverse commercial and research-grade immunoassays with variable specificity for KP-10 versus longer kisspeptin isoforms, inconsistent detection limits, and the absence of cross-platform calibration standards [25,26,92]. Pre-analytical variability—including sample matrix, processing delays, storage conditions, and protease inhibition—further compromises reproducibility. Development of isoform-specific, externally validated assays with certified reference materials and standardised pre-analytical protocols represents a prerequisite for meaningful inter-study comparison.

A second critical gap is the absence of robust gestational age–specific normative data. Although trimester-specific medians are frequently reported, large population-based reference intervals accounting for maternal BMI, ethnicity, smoking status, and metabolic comorbidities are lacking [26,90]. Given the exponential rise of KP-10 across gestation, failure to model gestational age appropriately introduces systematic bias. Future studies must employ gestational age-adjusted MoM and predefined confounder panels within multivariable frameworks.

Equally important is the need for precise phenotype definition. Early- and late-onset FGR and PE represent biologically distinct entities with differing placental pathology and vascular signatures. Most existing studies are underpowered for stratified analyses, limiting insight into phenotype-specific associations. Standardised diagnostic criteria and uniform Doppler-based classification must be incorporated prospectively.

The optimal design to address these gaps is a large, prospective, longitudinal cohort with serial biomarker sampling from the first trimester onwards. Such a study should include predefined primary endpoints (e.g., early-onset FGR < 32 weeks and preterm PE < 37 weeks) and external validation cohorts. Sample size calculations must ensure adequate power for phenotype-specific subgroup analyses. Beyond traditional discrimination metrics such as AUC, evaluation of incremental predictive value should incorporate net reclassification improvement (NRI), integrated discrimination improvement (IDI), calibration assessment, and decision-curve analysis to determine clinical net benefit. Only through these methodologies can the additive value of KP-10 within competing-risks models be rigorously assessed.

A harmonised minimal dataset is essential to ensure comparability across cohorts. This should include standardised maternal characteristics, mean arterial pressure, foetal biometry, comprehensive Doppler indices, pregnancy outcomes, and concurrent measurement of angiogenic biomarkers such as PlGF and sFlt-1 [1,117,118,119,120,121,122,123]. Uniform reporting standards aligned with international obstetric consensus statements are necessary to facilitate meta-analytic integration and external validation.

The most plausible clinical application for KP-10 lies within first-trimester multimarker screening models aimed at identifying pregnancies at risk of early placental dysfunction. Whether incorporation of KP-10 improves detection rates beyond current competing-risks frameworks remains an unresolved empirical question. Until standardised assays, gestational age-specific reference intervals, and prospective validation studies with formal incremental modelling are completed, KP-10 should be regarded as a promising investigational biomarker rather than a tool for routine clinical use.

In conclusion, the next phase of research must shift from exploratory association studies to validation-focused translational science. Only rigorously designed, adequately powered, and methodologically harmonised prospective cohorts will determine whether KP-10 contributes independently to precision obstetric risk stratification or remains primarily a mechanistic marker of placental biology.

## 9. Conclusions

KP-10 represents a biologically coherent marker of trophoblast signalling and early placental development, occupying an upstream position within the pathophysiological cascade of placental dysfunction. Accumulating molecular and clinical evidence supports its association with FGR and PE, particularly in early gestation. However, methodological heterogeneity, modest effect sizes, and lack of prospective validation currently limit its clinical applicability. Future research must transition from exploratory association studies to harmonised, validation-driven cohorts capable of determining whether KP-10 provides incremental predictive value within established multimarker screening models. Until such evidence is available, KP-10 should be regarded as a promising but investigational biomarker in the translational continuum of placental disease. Within the framework of molecular placental disease, the KISS1/KISS1R axis may represent a regulatory vulnerability node linking environmental stressors, epigenetic instability, and invasion-restrictive signalling.

## Figures and Tables

**Table 1:** Molecular mechanisms of KISS1/KISS1R signalling in placentation.

Pathway/Axis	Molecular Mediator	Cellular Effect	Placental Consequence	Key Evidence
Extracellular matrix remodelling	Downregulation of MMP-1, -2, -3, -7, -9, -10, and -14; upregulation of TIMP-1 and TIMP-3	Reduced trophoblast invasion and migration	Controlled spiral artery remodelling	[38,39]
ERK1/2 signalling	ERK1/2 phosphorylation	Regulation of invasion and angiogenesis gene expression	Fine-tuning of invasion depth	[38,42]
Integrin/adhesion signalling	PKC- and ERK1/2-dependent adhesion to collagen I	Increased extravillous trophoblast adhesion	Modulation of decidual anchoring	[42]
Angiogenic modulation	↓ VEGF-A; endothelial inhibition	Reduced endothelial proliferation and migration	Regulation of placental vascular development	[36,38]
TGF-β1 feedback	TGF-β1-induced KISS1 upregulation	Enhanced invasion suppression	Reinforcement of inhibitory control	[47]
Genetic ablation	Kiss1r knockout → ↑ Mmp2	Excessive invasion	Abnormal placental architecture	[32,41]
Early-onset PE dysregulation	↑ placental KISS1/KISS1R	Excessive inhibitory signalling	Shallow invasion and defective remodelling	[35,40]

KISS1, kisspeptin 1 gene; KISS1R, kisspeptin receptor; MMP, matrix metalloproteinase; TIMP, tissue inhibitor of metalloproteinase; ERK1/2, extracellular signal-regulated kinase 1/2; PKC, protein kinase C; VEGF-A, vascular endothelial growth factor A; PE, pre-eclampsia.

**Table 2:** Comparative diagnostic performance of kisspeptin and angiogenic biomarkers across gestation.

Gestational Window	Biomarker	Direction of Change in PE/FGR	Diagnostic Performance	Key References
First trimester (≤14 weeks)	Kisspeptin	Reduced in pregnancies later complicated by FGR/PE	AUC 0.80 (selected population); sensitivity 85.7%, specificity 71.4%	[92]
	PAPP-A	Reduced in PE/FGR	OR 2.08 (SGA), OR 1.94 (PE); LR+ 1.96; LR− 0.93	[95]
	PlGF	Reduced early in PE/FGR	Detection rate 56.3% at 10% SPR (with maternal factors); AUC 0.78–0.79	[93,94]
	PlGF + ADMA	Reduced PlGF, elevated ADMA	AUC 0.902 for PE	[96]
Second trimester (16–24 weeks)	Kisspeptin	Modest, phenotype-dependent	Greatest separation at 16–20 weeks; inconsistent thereafter	[91,97]
	PlGF ± Doppler	Reduced in placental disease	AUC 0.866 for adverse outcomes in early SGA	[98]
Third trimester (≥28 weeks)	Kisspeptin	Variable; lower in some FGR; higher in late PE	PPV 64.6%; NPV 87.5% (late FGR)	[91]
	sFlt-1/PlGF ratio	Elevated ratio in PE/FGR	AUC 0.92; ratio > 38: NPV 99.3%; ≥74: sensitivity 87.7%, specificity 97.0%	[99,100,101]
	PlGF alone	Reduced in PE	Sensitivity 96%; NPV 98% for PE requiring delivery ≤14 days	[102]

KP-10, kisspeptin-10; PE, pre-eclampsia; FGR, foetal growth restriction; SGA, small for gestational age; PAPP-A, pregnancy-associated plasma protein A; PlGF, placental growth factor; sFlt-1, soluble fms-like tyrosine kinase-1; ADMA, asymmetric dimethylarginine; AUC, area under the curve; OR, odds ratio; LR+, positive likelihood ratio; LR−, negative likelihood ratio; PPV, positive predictive value; NPV, negative predictive value; SPR, screen-positive rate.

**Table 3:** Sources of methodological heterogeneity.

Domain	Specific Source	Impact on Results	Standardisation Requirement	Key References
Analytical variability	Variable assays, isoform cross-reactivity and non-harmonised calibration	Non-comparable concentrations	Reference materials: isoform-specific reporting	[25,26,33,90]
Pre-analytical factors	Serum vs. plasma: delayed processing	Degradation bias	EDTA plasma; rapid processing	[25,26]
Gestational age	Exponential rise across gestation	Physiological misclassification	GA-adjusted models; MoM	[26,27,28,29]
Phenotype stratification	Early vs. late PE/FGR	Opposite directionality	Phenotype-specific analysis	[9,10,27,108]
Maternal confounders	BMI, insulin resistance, GDM	Confounding bias	Multivariable adjustment	[37,109,111]
Outcome definition	FGR vs. SGA misclassification	Diluted associations	Uniform criteria	[1,2,9]
Statistical design	Small cohorts; retrospective design	Limited generalisability	Prospective validation	[25,26,108]

## Data Availability

No new data were created or analyzed in this study. Data sharing is not applicable to this article.

## Abbreviations

The following abbreviations are used in this manuscript:

FGR	Foetal growth restriction
PE	Pre-eclampsia
KP-10	Kisspeptin-10
KISS1	Kisspeptin 1 gene
KISS1R	Kisspeptin 1 receptor
hCG	Human chorionic gonadotropin
PlGF	Placental growth factor
sFlt-1	Soluble fms-like tyrosine kinase-1
ELISA	Enzyme-linked immunosorbent assay
BMI	Body mass index
GA	Gestational age
EVT	Extravillous trophoblast
MAPK	Mitogen-activated protein kinase
PI3K	Phosphoinositide 3-kinase
AKT	Protein kinase B
mTOR	Mechanistic target of rapamycin
HIF	Hypoxia-inducible factor
VEGF	Vascular endothelial growth factor
NO	Nitric oxide

## References

[1] Martins J.G., Biggio J.R., Abuhamad A. (2020). Society for Maternal-Fetal Medicine Consult Series #52: Diagnosis and management of fetal growth restriction. Am. J. Obstet. Gynecol..

[2] (2021). Fetal growth restriction. Obstet. Gynecol..

[3] Tedyanto C.P., Prasetyadi F.O.H., Dewi S., Noorlaksmiatmo H. (2025). Maternal factors and perinatal outcomes associated with early-onset versus late-onset fetal growth restriction: A meta-analysis. J. Matern. Fetal Neonatal Med..

[4] Westby A., Miller L. (2021). Fetal growth restriction before and after birth. Am. Fam. Physician.

[5] Figueras F., Caradeux J., Crispi F., Eixarch E., Peguero A., Gratacos E. (2018). Diagnosis and surveillance of late-onset fetal growth restriction. Am. J. Obstet. Gynecol..

[6] McCowan L.M., Figueras F., Anderson N.H. (2018). Evidence-based national guidelines for the management of suspected fetal growth restriction: Comparison, consensus, and controversy. Am. J. Obstet. Gynecol..

[7] Lees C., Stampalija T., Hecher K. (2021). Diagnosis and management of fetal growth restriction: The ISUOG guideline and comparison with the SMFM guideline. Ultrasound Obstet. Gynecol..

[8] Figueras F., Gratacos E. (2016). An integrated approach to fetal growth restriction. Best Pract. Res. Clin. Obstet. Gynaecol..

[9] Lees C.C., Romero R., Stampalija T., Dall’Asta A., DeVore G.R., Prefumo F., Frusca T., Visser G.H.A., Hobbins J.C., Baschat A.A. (2022). The diagnosis and management of suspected fetal growth restriction: An evidence-based approach. Am. J. Obstet. Gynecol..

[10] Chappell L.C., Cluver C.A., Kingdom J., Tong S. (2021). Pre-eclampsia. Lancet.

[11] Burton G.J., Jauniaux E. (2018). Pathophysiology of placental-derived fetal growth restriction. Am. J. Obstet. Gynecol..

[12] Aplin J.D., Myers J.E., Timms K., Westwood M. (2020). Tracking placental development in health and disease. Nat. Rev. Endocrinol..

[13] Ives C.W., Sinkey R., Rajapreyar I., Tita A.T.N., Oparil S. (2020). Preeclampsia—Pathophysiology and clinical presentations. J. Am. Coll. Cardiol..

[14] Burton G.J., Yung H.-W., Cindrova-Davies T., Charnock-Jones D.S. (2008). Placental endoplasmic reticulum stress and oxidative stress in the pathophysiology of unexplained intrauterine growth restriction and early onset preeclampsia. Placenta.

[15] Rana S., Burke S.D., Karumanchi S.A. (2020). Imbalances in circulating angiogenic factors in the pathophysiology of preeclampsia and related disorders. Am. J. Obstet. Gynecol..

[16] Mecacci F., Avagliano L., Lisi F., Clemenza S., Serena C., Vannuccini S., Rambaldi M.P., Simeone S., Ottanelli S., Petraglia F. (2020). Fetal Growth restriction: Does an Integrated Maternal Hemodynamic-Placental model fit better?. Reprod. Sci..

[17] Stepan H., Hund M., Andraczek T. (2020). Combining biomarkers to predict pregnancy complications and redefine preeclampsia. Hypertension.

[18] Stepan H., Galindo A., Hund M., Schlembach D., Sillman J., Surbek D., Vatish M. (2022). Clinical utility of sFlt-1 and PlGF in screening, prediction, diagnosis and monitoring of pre-eclampsia and fetal growth restriction. Ultrasound Obstet. Gynecol..

[19] Silasi M., Azzi M., Potchileev S., Burns L., Rana S. (2025). Placental biomarker testing for evaluation of suspected preeclampsia. Clin. Chem..

[20] Youssef L., Crispi F., Paolucci S., Miranda J., Lobmaier S., Crovetto F., Figueras F., Gratacos E. (2025). Angiogenic factors alone or in combination with ultrasound Doppler criteria for risk classification among late-onset small fetuses with or without pre-eclampsia. Ultrasound Obstet. Gynecol..

[21] Hong J., Crawford K., Daly M., Clifton V., Da Silva Costa F., Perkins A.V., Matsika A., Lourie R., Kumar S. (2024). Utility of placental biomarkers and fetoplacental Dopplers in predicting likely placental pathology in early and late fetal growth restriction—A prospective study. Placenta.

[22] Villalaín C., Galindo A., D’Antonio F., Herraiz I. (2025). Clinical use of angiogenesis biomarkers in fetal growth restriction: A narrative review. J. Matern. Fetal Neonatal Med..

[23] Chaemsaithong P., Gil M.M., Chaiyasit N., Cuenca-Gomez D., Plasencia W., Rolle V., Poon L.C. (2023). Accuracy of placental growth factor alone or in combination with soluble fms-like tyrosine kinase-1 or maternal factors in detecting preeclampsia in asymptomatic women in the second and third trimesters: A systematic review and meta-analysis. Am. J. Obstet. Gynecol..

[24] Tsoutsouki J., Patel B., Comninos A.N., Dhillo W.S., Abbara A. (2022). Kisspeptin in the prediction of pregnancy complications. Front. Endocrinol..

[25] Hu K.-L., Zhao H., Yu Y., Li R. (2019). Kisspeptin as a potential biomarker throughout pregnancy. Eur. J. Obstet. Gynecol. Reprod. Biol..

[26] Abbara A., Al-Memar M., Phylactou M., Daniels E., Patel B., Eng P.C., Nadir R., Izzi-Engbeaya C., Clarke S.A., Mills E.G. (2021). Changes in circulating kisspeptin levels during each trimester in women with antenatal complications. J. Clin. Endocrinol. Metab..

[27] Jayasena C.N., Abbara A., Izzi-Engbeaya C., Comninos A.N., Harvey R.A., Maffe J.G., Sarang Z., Ganiyu-Dada Z., Padilha A.I., Dhanjal M. (2014). Reduced levels of plasma kisspeptin during the antenatal booking visit are associated with increased risk of miscarriage. J. Clin. Endocrinol. Metab..

[28] Ibanoglu M.C., Oskovi-Kaplan Z.A., Kara O., Ozgu-Erdinc A.S., Şahin D. (2023). Relationship between kisspeptin levels in the third trimester and late-onset fetal growth restriction: A case-control study. Placenta.

[29] Hu K.-L., Chang H.-M., Zhao H.-C., Yu Y., Li R., Qiao J. (2018). Potential roles for the kisspeptin/kisspeptin receptor system in implantation and placentation. Hum. Reprod. Update.

[30] Radovick S., Babwah A.V. (2019). Regulation of Pregnancy: Evidence for major roles by the uterine and placental Kisspeptin/KISS1R signaling systems. Semin. Reprod. Med..

[31] Baethge C., Goldbeck-Wood S., Mertens S. (2019). SANRA—A Scale for the Quality Assessment of Narrative Review Articles. Res. Integr. Peer Rev..

[32] Panting E.N., Weight J.H., Sartori J.A., Coall D.A., Smith J.T. (2024). The role of placental kisspeptin in trophoblast invasion and migration: An assessment in Kiss1r knockout mice, BeWo cell lines and human term placenta. Reprod. Fertil. Dev..

[33] Chakraborty A.P., Banerjee A.A. (2025). Kisspeptin isoforms: Versatile players in reproduction and beyond. J. Mol. Endocrinol..

[34] Mead E.J., Maguire J.J., Kuc R.E., Davenport A.P. (2007). Kisspeptins: A multifunctional peptide system with a role in reproduction, cancer and the cardiovascular system. Br. J. Pharmacol..

[35] Cartwright J.E., Williams P.J. (2012). Altered placental expression of kisspeptin and its receptor in pre-eclampsia. J. Endocrinol..

[36] Ramaesh T., Logie J.J., Roseweir A.K., Millar R.P., Walker B.R., Hadoke P.W.F., Reynolds R.M. (2010). Kisspeptin-10 Inhibits Angiogenesis in Human Placental Vessels ex Vivo and Endothelial Cells in Vitro. Endocrinology.

[37] Musa E., Salazar-Petres E., Vatish M., Levitt N., Sferruzzi-Perri A.N., Matjila M.J. (2024). Kisspeptin signalling and its correlation with placental ultrastructure and clinical outcomes in pregnant South African women with obesity and gestational diabetes. Placenta.

[38] Francis V.A., Abera A.B., Matjila M., Millar R.P., Katz A.A. (2014). Kisspeptin regulation of genes involved in cell invasion and angiogenesis in first trimester human trophoblast cells. PLoS ONE.

[39] Bilban M., Ghaffari-Tabrizi N., Hintermann E., Bauer S., Molzer S., Zoratti C., Malli R., Sharabi A., Hiden U., Graier W. (2004). Kisspeptin-10, a KiSS-1/metastin-derived decapeptide, is a physiological invasion inhibitor of primary human trophoblasts. J. Cell Sci..

[40] Qiao C., Wang C., Zhao J., Liu C., Shang T. (2012). Elevated Expression of KiSS-1 in Placenta of Chinese Women with Early-Onset Preeclampsia. PLoS ONE.

[41] Decourt C., Heads K. (2025). Kisspeptin and early pregnancy: Insights from animal models into hormonal regulation and miscarriage. J. Neuroendocr..

[42] Taylor J., Pampillo M., Bhattacharya M., Babwah A.V. (2013). Kisspeptin/KISS1R signaling potentiates extravillous trophoblast adhesion to type-I collagen in a PKC- and ERK1/2-dependent manner. Mol. Reprod. Dev..

[43] Szydełko-Gorzkowicz M., Poniedziałek-Czajkowska E., Mierzyński R., Sotowski M., Leszczyńska-Gorzelak B. (2022). The Role of Kisspeptin in the Pathogenesis of Pregnancy Complications: A Narrative Review. Int. J. Mol. Sci..

[44] Babwah A.V., Pampillo M., Min L., Kaiser U.B., Bhattacharya M. (2012). Single-Cell Analyses Reveal That KISS1R-Expressing Cells Undergo Sustained Kisspeptin-Induced Signaling That Is Dependent upon An Influx of Extracellular Ca^2+^. Endocrinology.

[45] Castaño J.P., Martínez-Fuentes A.J., Gutiérrez-Pascual E., Vaudry H., Tena-Sempere M., Malagón M.M. (2008). Intracellular signaling pathways activated by kisspeptins through GPR54: Do multiple signals underlie function diversity?. Peptides.

[46] Roseweir A.K., Katz A.A., Millar R.P. (2012). Kisspeptin-10 inhibits cell migration in vitro via a receptor-GSK3 beta-FAK feedback loop in HTR8SVneo cells. Placenta.

[47] Fang L., Yan Y., Gao Y., Wu Z., Wang Z., Yang S., Cheng J.-C., Sun Y.-P. (2022). TGF-β1 inhibits human trophoblast cell invasion by upregulating kisspeptin expression through ERK1/2 but not SMAD signaling pathway. Reprod. Biol. Endocrinol..

[48] Matjila M., Millar R., Van Der Spuy Z., Katz A. (2013). The Differential Expression of Kiss1, MMP9 and Angiogenic Regulators across the Feto-Maternal Interface of Healthy Human Pregnancies: Implications for Trophoblast Invasion and Vessel Development. PLoS ONE.

[49] Huang Z., Huang S., Song T., Yin Y., Tan C. (2021). Placental Angiogenesis in Mammals: A Review of the Regulatory Effects of Signaling Pathways and Functional Nutrients. Adv. Nutr..

[50] Geva E., Ginzinger D.G., Zaloudek C.J., Moore D.H., Byrne A., Jaffe R.B. (2002). Human Placental Vascular Development: Vasculogenic and Angiogenic (Branching and Nonbranching) Transformation Is Regulated by Vascular Endothelial Growth Factor-A, Angiopoietin-1, and Angiopoietin-2. J. Clin. Endocrinol. Metab..

[51] Burton G.J., Charnock-Jones D.S., Jauniaux E. (2009). Regulation of vascular growth and function in the human placenta. Reproduction.

[52] Zygmunt M., Herr F., Münstedt K., Lang U., Liang O.D. (2003). Angiogenesis and vasculogenesis in pregnancy. Eur. J. Obstet. Gynecol. Reprod. Biol..

[53] Kaufmann P., Mayhew T.M., Charnock-Jones D.S. (2003). Aspects of human fetoplacental vasculogenesis and angiogenesis. II. Changes during normal pregnancy. Placenta.

[54] Umapathy A., Chamley L.W., James J.L. (2019). Reconciling the distinct roles of angiogenic/anti-angiogenic factors in the placenta and maternal circulation of normal and pathological pregnancies. Angiogenesis.

[55] Pang V., Bates D.O., Leach L. (2017). Regulation of human feto-placental endothelial barrier integrity by vascular endothelial growth factors: Competitive interplay between VEGF-A165a, VEGF-A165b, PIGF and VE-cadherin. Clin. Sci..

[56] Wulff C., Weigand M., Kreienberg R., Fraser H.M. (2003). Angiogenesis during primate placentation in health and disease. Reproduction.

[57] Alfaidy N., Brouillet S., Rajaraman G., Kalionis B., Hoffmann P., Barjat T., Benharouga M., Murthi P. (2020). The Emerging Role of the Prokineticins and Homeobox Genes in the Vascularization of the Placenta: Physiological and Pathological Aspects. Front. Physiol..

[58] Reynolds L.P., Redmer D.A. (2001). Angiogenesis in the Placenta1. Biol. Reprod..

[59] Wang K., Zheng J. (2011). Signaling regulation of fetoplacental angiogenesis. J. Endocrinol..

[60] Esposito M., Paulesu L., Mandalà M. (2025). The role of placental hormones and metabolites in modulating uterine circulation in physiological and pathological pregnancies. Front. Endocrinol..

[61] Yang Y., Zhang S., Fang X., Shao T., Shao X., Wang Y.-L. (2025). Placental Steroid Hormones in Preeclampsia: Multilayered Regulation of Endocrine Pathogenesis. Endocr. Rev..

[62] Chiang Y.-T., Seow K.-M., Chen K.-H. (2024). The Pathophysiological, Genetic, and Hormonal Changes in Preeclampsia: A Systematic Review of the Molecular Mechanisms. Int. J. Mol. Sci..

[63] Aye I.L.M.H., Aiken C.E., Charnock-Jones D.S., Smith G.C.S. (2020). Placental energy metabolism in health and disease—Significance of development and implications for preeclampsia. Am. J. Obstet. Gynecol..

[64] Hu M., Li J., Baker P.N., Tong C. (2021). Revisiting preeclampsia: A metabolic disorder of the placenta. FEBS J..

[65] De Knegt V.E., Hedley P.L., Kanters J.K., Thagaard I.N., Krebs L., Christiansen M., Lausten-Thomsen U. (2021). The Role of Leptin in Fetal Growth during Pre-Eclampsia. Int. J. Mol. Sci..

[66] Gennari-Moser C., Khankin E.V., Schüller S., Escher G., Frey B.M., Portmann C.-B., Baumann M.U., Lehmann A.D., Surbek D., Karumanchi S.A. (2010). Regulation of Placental Growth by Aldosterone and Cortisol. Endocrinology.

[67] Ness R.B., Sibai B.M. (2006). Shared and disparate components of the pathophysiologies of fetal growth restriction and preeclampsia. Am. J. Obstet. Gynecol..

[68] Yagel S., Cohen S.M., Goldman-Wohl D. (2021). An integrated model of preeclampsia: A multifaceted syndrome of the maternal cardiovascular-placental-fetal array. Am. J. Obstet. Gynecol..

[69] Mark P.J., Jones M.L., Lewis J.L., Waddell B.J., Smith J.T. (2013). Kiss1 and Kiss1r mRNA expression in the rat placenta: Changes with gestational age and regulation by glucocorticoids. Placenta.

[70] Zhang B., Kim M.Y., Elliot G., Zhou Y., Zhao G., Li D., Lowdon R.F., Gormley M., Kapidzic M., Robinson J.F. (2021). Human placental cytotrophoblast epigenome dynamics over gestation and alterations in placental disease. Dev. Cell.

[71] Chen Y., Ye Z., Lin M., Zhu L., Xu L., Wang X. (2024). Deciphering the epigenetic landscape: Placental development and its role in pregnancy outcomes. Stem Cell Rev. Rep..

[72] Januar V., Desoye G., Novakovic B., Cvitic S., Saffery R. (2015). Epigenetic regulation of human placental function and pregnancy outcome: Considerations for causal inference. Am. J. Obstet. Gynecol..

[73] Semaan S.J., Kauffman A.S. (2013). Emerging concepts on the epigenetic and transcriptional regulation of the Kiss1 gene. Int. J. Dev. Neurosci..

[74] Kapustin R.V., Drobintseva A.O., Alekseenkova E.N., Onopriychuk A.R., Arzhanova O.N., Polyakova V.O., Kvetnoy I.M. (2019). Placental protein expression of kisspeptin-1 (KISS1) and the kisspeptin-1 receptor (KISS1R) in pregnancy complicated by diabetes mellitus or preeclampsia. Arch. Gynecol. Obstet..

[75] Kariori M., Katsi V., Tsioufis C. (2025). Late vs. Early Preeclampsia. Int. J. Mol. Sci..

[76] Spinillo A., Gardella B., Adamo L., Muscettola G., Fiandrino G., Cesari S. (2019). Pathologic placental lesions in early and late fetal growth restriction. Acta Obstet. Gynecol. Scand..

[77] Mifsud W., Sebire N.J. (2014). Placental Pathology in Early-Onset and Late-Onset Fetal Growth Restriction. Fetal Diagn. Ther..

[78] Solt I., Cohen S.M., Admati I., Beharier O., Dominsky O., Yagel S. (2025). Placenta at single-cell resolution in early and late preeclampsia: Insights and clinical implications. Am. J. Obstet. Gynecol..

[79] Burton G.J., Redman C.W., Roberts J.M., Moffett A. (2019). Pre-eclampsia: Pathophysiology and clinical implications. BMJ.

[80] Magee L.A., Nicolaides K.H., Von Dadelszen P. (2022). Preeclampsia. N. Engl. J. Med..

[81] Mecacci F., Romani E., Clemenza S., Zullino S., Avagliano L., Petraglia F. (2023). Early Fetal Growth Restriction with or Without Hypertensive Disorders: A Clinical Overview. Reprod. Sci..

[82] Kingdom J.C., Audette M.C., Hobson S.R., Windrim R.C., Morgen E. (2017). A placenta clinic approach to the diagnosis and management of fetal growth restriction. Am. J. Obstet. Gynecol..

[83] Muniz C.S., Dias B.F., Motoyama P.V.P., Almeida C.T.C., De Lucena Feitosa F.E., Júnior E.A., Alves J.A.G. (2021). Doppler abnormalities and perinatal outcomes in pregnant women with early-onset fetal growth restriction. J. Matern. Fetal Neonatal Med..

[84] ACOG Fetal Growth Restriction. https://www.acog.org/clinical/clinical-guidance/practice-bulletin/articles/2021/02/fetal-growth-restriction.

[85] Berkley E., Chauhan S.P., Abuhamad A. (2012). Doppler assessment of the fetus with intrauterine growth restriction. Am. J. Obstet. Gynecol..

[86] Tay J., Masini G., McEniery C.M., Giussani D.A., Shaw C.J., Wilkinson I.B., Bennett P.R., Lees C.C. (2018). Uterine and fetal placental Doppler indices are associated with maternal cardiovascular function. Am. J. Obstet. Gynecol..

[87] Marijnen M.C., Van Der Meeren L.E., Schoots M.H., Freedman A.A., Ernst L.M., Bazyleva E., Ganzevoort W., Gordijn S.J., Schaaf J.M., De Boer M.A. (2025). Placental lesions in small for gestational age fetuses with and without clinical features of fetal growth restriction: A secondary analysis of the Doppler Ratio In fetal Growth restriction Intervention Trial At (near) Term (DRIGITAT) study. Am. J. Obstet. Gynecol..

[88] Gomes V.C.L., Sones J.L. (2021). From inhibition of trophoblast cell invasion to proapoptosis: What are the potential roles of kisspeptins in preeclampsia?. Am. J. Physiol. Regul. Integr. Comp. Physiol..

[89] Armstrong R.A., Reynolds R.M., Leask R., Shearing C.H., Calder A.A., Riley S.C. (2009). Decreased serum levels of kisspeptin in early pregnancy are associated with intra-uterine growth restriction and pre-eclampsia. Prenat. Diagn..

[90] Pérez-López F.R., López-Baena M.T., Varikasuvu S.R., Ruiz-Román R., Fuentes-Carrasco M., Savirón-Cornudella R. (2021). Preeclampsia and gestational hypertension are associated to low maternal circulating kisspeptin levels: A systematic review and meta-analysis. Gynecol. Endocrinol..

[91] Ibanoglu M.C., Oskovi-Kaplan Z.A., Ozgu-Erdinc A.S., Kara O., Sahin D. (2022). Comparison of the Kisspeptin levels in early onset preeclampsia and late-onset preeclampsia. Arch. Gynecol. Obstet..

[92] Logie J.J., Denison F.C., Riley S.C., Ramaesh T., Forbes S., Norman J.E., Reynolds R.M. (2011). Evaluation of kisspeptin levels in obese pregnancy as a biomarker for pre-eclampsia. Clin. Endocrinol..

[93] Rode L., Wright A., Wright D., Overgaard M., Sperling L., Sandager P., Nørgaard P., Jørgensen F.S., Zingenberg H., Riishede I. (2025). Screening for pre-eclampsia using pregnancy-associated plasma protein-A or placental growth factor measurements in blood samples collected at 8–14 weeks’ gestation. Ultrasound Obstet. Gynecol..

[94] Noël L., Guy G.P., Jones S., Forenc K., Buck E., Papageorghiou A.T., Thilaganathan B. (2021). Routine first-trimester combined screening for pre-eclampsia: Pregnancy-associated plasma protein-A or placental growth factor?. Ultrasound Obstet. Gynecol..

[95] Morris R.K., Bilagi A., Devani P., Kilby M.D. (2016). Association of serum PAPP-A levels in first trimester with small for gestational age and adverse pregnancy outcomes: Systematic review and meta-analysis. Prenat. Diagn..

[96] Bian Z., Shixia C., Duan T. (2015). First-Trimester maternal serum levels of SFLT1, PGF and ADMA predict preeclampsia. PLoS ONE.

[97] Abbara A., Al-Memar M., Phylactou M., Kyriacou C., Eng P.C., Nadir R., Izzi-Engbeaya C., Clarke S.A., Mills E.G., Daniels E. (2021). Performance of plasma kisspeptin as a biomarker for miscarriage improves with gestational age during the first trimester. Fertil. Steril..

[98] Bonacina E., Armengol-Alsina M., Casellas A., Dalmau M., Diaz P., Roldán E., Temprado J., Duaso M., Ampurdanes Q., José M.S. (2026). Angiogenic factors and fetal Doppler for predicting adverse pregnancy outcome in early-onset small fetuses with and without pre-eclampsia. Ultrasound Obstet. Gynecol..

[99] Berg A.H., Calsavara V.F., Hund M., Guo G., Rana S., Costantine M.M., Espinoza J., Boggess K., Wylie B.J., Simas T.A.M. (2025). Serum soluble-fms-like tyrosine kinase 1-to-placental growth factor ratio on Elecsys immunoassay platform predicts preeclampsia with severe features in hospitalized women with hypertensive disorders of pregnancy. Am. J. Obstet. Gynecol..

[100] Pan X., Peng J., Chen Y., Di X., Li P., Zhang G., Liu H. (2025). sFlt-1/ PlGF ratio thresholds for diagnosing pre-eclampsia in pregnant women with high blood pressure. Ultrasound Obstet. Gynecol..

[101] Agrawal S., Cerdeira A.S., Redman C., Vatish M. (2017). Meta-Analysis and systematic review to assess the role of soluble FMS-Like tyrosine kinase-1 and placenta growth factor ratio in prediction of preeclampsia. Hypertension.

[102] Duhig K.E., Myers J., Seed P.T., Sparkes J., Lowe J., Hunter R.M., Shennan A.H., Chappell L.C., Bahl R., Bambridge G. (2019). Placental growth factor testing to assess women with suspected pre-eclampsia: A multicentre, pragmatic, stepped-wedge cluster-randomised controlled trial. Lancet.

[103] Byrne Z., Brennan S., Watson D., Rudd D., Kandasamy Y. (2026). Maternal SFLT-1/ PLGF Ratio to Distinguish Pathological fetal growth restriction from constitutional smallness: Systematic review. Int. J. Obstet. Gynaecol..

[104] Maurer J., Grouzmann E., Eugster P.J. (2023). Tutorial review for peptide assays: An ounce of pre-analytics is worth a pound of cure. J. Chromatogr. B Anal. Technol. Biomed. Life Sci..

[105] Rai A.J., Vitzthum F. (2006). Effects of preanalytical variables on peptide and protein measurements in human serum and plasma: Implications for clinical proteomics. Expert Rev. Proteom..

[106] Liu Z., Ren C., Jones W., Chen P., Seminara S.B., Chan Y.-M., Smith N.F., Covey J.M., Wang J., Chan K.K. (2013). LC–MS/MS quantification of a neuropeptide fragment kisspeptin-10 (NSC 741805) and characterization of its decomposition product and pharmacokinetics in rats. J. Chromatogr. B Anal. Technol. Biomed. Life Sci..

[107] Sullivan-Pyke C., Haisenleder D.J., Senapati S., Nicolais O., Eisenberg E., Sammel M.D., Barnhart K.T. (2018). Kisspeptin as a new serum biomarker to discriminate miscarriage from viable intrauterine pregnancy. Fertil. Steril..

[108] Medina A.C., Gómez-Acebo I., De Largy C.C.G., Alonso-Molero J., Calvo S.V., Feu J.M.O., Medina M.C., Dierssen-Sotos T. (2025). Study of first-trimester serum levels of β-hCG and PAPP-A as a screening test for fetal development of intrauterine growth restriction. BMC Pregnancy Childbirth.

[109] Sitticharoon C., Mutirangura P., Chinachoti T., Iamaroon A., Triyasunant N., Churintaraphan M., Keadkraichaiwat I., Maikaew P., Sririwichitchai R. (2020). Associations of serum kisspeptin levels with metabolic and reproductive parameters in men. Peptides.

[110] Jayasena C.N., Comninos A.N., Narayanaswamy S., Abbara A., Nijher G.M., Cheema M., Malik Z., Ghatei M.A., Bloom S.R., Dhillo W.S. (2014). The identification of elevated urinary kisspeptin-immunoreactivity during pregnancy. Ann. Clin. Biochem..

[111] Gao M., Tao X., Zhang Q., He W., Zhao T., Yuan T. (2023). Correlation between kisspeptin and biochemical markers in obese and non-obese women with polycystic ovary syndrome. Gynecol. Endocrinol..

[112] Ling B., Zhang X. (2025). Assessing placental endocrine and vascular function for prenatal prediction of adverse pregnancy outcomes in advanced maternal age pregnancies. J. Matern. Fetal Neonatal Med..

[113] Hong J., Kumar S. (2023). Circulating biomarkers associated with placental dysfunction and their utility for predicting fetal growth restriction. Clin. Sci..

[114] Van De Meent M., Schuit E., Ganzevoort W., Al-Nasiry S., Bekker M.N., Derks J.B., Duvekot J.J., Gordijn S.J., Groenendaal F., Jellema R. (2025). Temporal Changes in Fetal and maternal parameters in Early-Onset Fetal Growth Restriction: A Multicenter, Retrospective Cohort study. Int. J. Obstet. Gynaecol..

[115] Aviram A., Sherman C., Kingdom J., Zaltz A., Barrett J., Melamed N. (2018). Defining early vs. late fetal growth restriction by placental pathology. Acta Obstet. Gynecol. Scand..

[116] Fan H., Li L., Hao C. (2024). Clinical significance of three-dimensional power Doppler combined with two-dimensional Doppler ultrasonography for evaluating fetal growth restriction. J. Matern. Fetal Neonatal Med..

[117] Arechvo A., Wright A., Recalde A.N., Liandro R., Charakida M., Nicolaides K.H. (2023). Ophthalmic artery Doppler and biomarkers of impaired placentation at 36 weeks’ gestation in pregnancies with small fetuses. Ultrasound Obstet. Gynecol..

[118] Khalil A., Gordijn S.J., Beune I.M., Wynia K., Ganzevoort W., Figueras F., Kingdom J., Marlow N., Papageorghiou A.T., Sebire N. (2018). Essential variables for reporting research studies on fetal growth restriction: A Delphi consensus. Ultrasound Obstet. Gynecol..

[119] Roberts L.A., Ling H.Z., Poon L.C., Nicolaides K.H., Kametas N.A. (2018). Maternal hemodynamics, fetal biometry and Doppler indices in pregnancies followed up for suspected fetal growth restriction. Ultrasound Obstet. Gynecol..

[120] Lopian M., Prasad S., Segal E., Dotan A., Ulusoy C.O., Khalil A. (2025). Prediction of small-for-gestational age and fetal growth restriction at routine ultrasound examination at 35–37 weeks’ gestation. Ultrasound Obstet. Gynecol..

[121] Meler E., Martínez J., Boada D., Mazarico E., Figueras F. (2021). Doppler studies of placental function. Placenta.

[122] Agrawal S., Parks W.T., Zeng H.D., Ravichandran A., Ashwal E., Windrim R.C., Hobson S.R., Melamed N., Kingdom J.C. (2022). Diagnostic utility of serial circulating placental growth factor levels and uterine artery Doppler waveforms in diagnosing underlying placental diseases in pregnancies at high risk of placental dysfunction. Am. J. Obstet. Gynecol..

[123] Lai J., Syngelaki A., Nicolaides K.H., Von Dadelszen P., Magee L.A. (2022). Using ultrasound and angiogenic markers from a 19- to 23-week assessment to inform the subsequent diagnosis of preeclampsia. Am. J. Obstet. Gynecol..

